# Fabrication and Characterization of Fully Inkjet Printed Capacitors Based on Ceramic/Polymer Composite Dielectrics on Flexible Substrates

**DOI:** 10.1038/s41598-019-49639-3

**Published:** 2019-09-16

**Authors:** Morten Mikolajek, Timo Reinheimer, Nicole Bohn, Christian Kohler, Michael J. Hoffmann, Joachim R. Binder

**Affiliations:** Institute for Applied Materials, Karlsruhe Institute of Technology, Hermann-von-Helmholtz-Platz 1, 76344 Eggenstein-Leopoldshafen, Germany

**Keywords:** Nanoscience and technology, Materials science

## Abstract

The preparation of fully inkjet printed capacitors containing ceramic/polymer composites as the dielectric material is presented. Therefore, ceramic/polymer composite inks were developed, which allow a fast one-step fabrication of the composite thick films. Ba_0.6_Sr_0.4_TiO_3_ (BST) is used as the ceramic component and poly(methyl methacrylate) (PMMA) as the polymer. The use of such composites allows printing on flexible substrates. Furthermore, it results in improved values for the permittivity compared to pure polymers. Three composite inks with varying ratio of BST to PMMA were used for the fabrication of composite thick films consisting of 33, 50 and 66 vol% BST, respectively. All inks lead to homogeneous structures with precise transitions between the different layers in the capacitors. Besides the microstructures of the printed thick films, the dielectric properties were characterized by impedance spectroscopy over a frequency range of 100 Hz to 200 kHz. In addition, the influence of a larger ceramic particle size was investigated, to raise permittivity. The printed capacitors exhibited dielectric constants of 20 up to 55 at 1 kHz. Finally, the experimental results were compared to different theoretical models and their suitability for the prediction of *ε*_composite_ was assessed.

## Introduction

In the late 1990s, the first successes of printed functional devices established the contemporary understanding of printed electronics, containing conductors, insulators and semiconductors^1–3^. Out of several printing technologies, especially the digital techniques like inkjet printing and in recent years various 3D-printing techniques pushed the development of printed electronics forward^4^. This is because digital techniques offer several advantages compared to traditional printing technologies like screen-printing. The digital and contactless method allows fabrication directly from a digital model, which leads to an enormous increase in flexibility regarding substrate, material and structural design as well as in cost and waste reduction^5,6^. Inkjet printing, with its origins in the graphics sector, is the most promising technology with regard to the fabrication of 2D functional layers, as it can be used for all relevant material classes, such as ceramics, polymers, metals and carbon nanotubes^7–10^.

First, to deposit a material with drop-on-demand (DOD) inkjet printing, it has to be dispersed or dissolved in a solvent to get the so-called ink. Such inks have to fulfill several requirements related to the particle size, the ink stability and the fluid mechanical properties, for getting a good jettability^11^. In addition, the major challenge of the inkjet printing technology is the control of the drying behavior of the inks on the substrate to obtain homogeneous structures. Printable inks have a low viscosity and a rather small solid content. Hence, they are usually subject to drying effects, of which the so-called coffee stain effect (CSE) is the most common. This effect, which leads to ring like structures, was first described by Deegan *et al*. and is since then an intensive research objective^12,13^. There are several approaches to prevent the CSE, like the use of the Marangoni effect, electrowetting, moving contact line, shape-dependent capillary interactions or increasing viscosity^14–19^. Nonetheless, all approaches require a detailed ink development adjusted to the used solvents and materials, which is the major drawback of the inkjet printing technology to date.

Looking at the recent developments concerning inkjet printing of passive electronic components, such as capacitors or resistors, two main areas can be identified. On the one hand, fabrication of pure ceramic dielectrics for specialized applications, such as tunable microwave components^20,21^. On the other hand, there are various polymer based inks for the fabrication of flexible or stretchable devices, because they need no high temperature sintering process and therefore can be used with polymer substrates^22–24^. Nonetheless, polymers only offer low values for their relative permittivity *ε*_r_ in the range of about 2 to 4^25^. To overcome this limitation it is common to add high-*ε* ceramic fillers to the polymer, such as barium titanate (BT) or barium strontium titanate (BST)^26–29^. This allows an increase of the permittivity, but at the same time no high temperature treatment is needed and polymer substrates can be used. While such composites are widely used in other applications they are surprisingly underrepresented in the area of inkjet printed dielectrics. Lim *et al*. showed the fabrication of semi-printed BaTiO_3_-resin capacitors with promising dielectric properties, but used an infiltration technique for the composite layer, which resulted in a thick layer of 20 microns on a non-flexible substrate^30,31^. Besides that, Kang *et al*. as well as Mikolajek *et al*. reported the fabrication of fully printed capacitors on flexible substrates using a composite ink, but both with only low ceramic content and thus low permittivity^32,33^. As they have shown, it will be crucial to establish composite inks in the inkjet technology as only composite inks can provide a fast and simple one-step fabrication.

When composite materials are used as the dielectric material it is very beneficial to precisely predict the permittivity of the composite system. Therefore, several theoretical models were proposed in the literature to describe the dielectric properties of two component systems. One of the simplest models is the description as a 2–2 composite type^34^. Then the permittivity can be defined as a function of the ceramic content using a circuit consisting of serial capacitors (Eq. 1) where *ε*_c_ and *φ*_c_ are the dielectric constant and the volume content of the ceramic component, *ε*_p_ is the dielectric constant of the polymer and *ε*_eff_ is the effective permittivity of the composite^35^.

1 $${\varepsilon }_{eff}=\frac{{\varepsilon }_{c}{\varepsilon }_{p}}{{\varepsilon }_{c}-{{\rm{\phi }}}_{c}{\varepsilon }_{c}+{{\rm{\phi }}}_{c}{\varepsilon }_{p}}$$

However, this model is unable to predict the dielectric properties of a printed particle based ceramic/polymer composite adequately, since both components are deposited together in the ink and no layered structure with isolated ceramic and polymer parts is received, as is assumed in the model^35^. In fact, the BST ceramic powders are assumed to be nearly spherical and uniformly distributed in the polymer matrix. Hence, 0–3 type composite models are more suitable for the printed composites discussed in this paper, as they predict that one dielectric material is distributed randomly in the other^35^. Due to the variety of models, only those of Lichtenecker, Jayasundere, Sherman and Looyenga will be considered here as examples. All of them showed their suitability for the prediction of the dielectric composite properties in experimental comparisons. Lichtenecker weighted the permittivity of the individual components logarithmically (Eq. 2)^36^. Although it is based on 1–3 connectivity, it has often shown its applicability and is therefore often used in the literature^37,38^.

2 $$\log \,{\varepsilon }_{eff}={{\rm{\phi }}}_{p}\,\log \,{\varepsilon }_{p}+{{\rm{\phi }}}_{c}\,\log \,{\varepsilon }_{c}$$

Jayasundere and Smith developed a model for binary piezoelectric composites by modifying the Kerner equation^39^. They proceed from a significant difference in the permittivity of the particles and the matrix and therefore involve interactions between adjacent particles in the calculation (Eq. 3)^40^.

3 $${\varepsilon }_{eff}=\frac{{\phi }_{p}{\varepsilon }_{p}+{\phi }_{c}{\varepsilon }_{c}[\frac{3{\varepsilon }_{p}}{{\varepsilon }_{c}+2{\varepsilon }_{p}}][1+\frac{3{\phi }_{c}({\varepsilon }_{c}-{\varepsilon }_{p})}{{\varepsilon }_{c}+2{\varepsilon }_{p}}]}{{\phi }_{p}+{\phi }_{c}[\frac{3{\varepsilon }_{p}}{{\varepsilon }_{c}+2{\varepsilon }_{p}}][1+\frac{3{\phi }_{c}({\varepsilon }_{c}-{\varepsilon }_{p})}{{\varepsilon }_{c}+2{\varepsilon }_{p}}]}$$

Based on the theory of “effective medium approximation” according to Bruggeman^41^, which already makes reliable calculations possible^42^, Sherman developed another model, where the complete miscibility of the ferroelectric (ceramic) and the dielectric (polymer) phase (Eq. 4) is included^43^.

4 $${\varepsilon }_{eff}=\frac{1}{4}\,[{\varepsilon }_{p}+3{\phi }_{p}{\varepsilon }_{p}+2{\varepsilon }_{c}-3{\phi }_{p}{\varepsilon }_{c}+\,\sqrt{8{\varepsilon }_{p}{\varepsilon }_{c}+{(-{\varepsilon }_{p}+3{\phi }_{p}{\varepsilon }_{p}+2{\varepsilon }_{c}-3{\phi }_{p}{\varepsilon }_{c})}^{2}}]$$

Looyenga provided another model, which is based on the considerations of Bruggeman. By eliminating the particle shape and possible interactions, it delivers a simple formula that is suitable for homogeneous mixtures (Eq. 5). The shape and permittivity of the particles are not exactly defined^44^.

5 $${\varepsilon }_{eff}={[({\varepsilon }_{c}^{\frac{1}{3}}-{\varepsilon }_{p}^{\frac{1}{3}}){{\rm{\phi }}}_{c}+{\varepsilon }_{p}^{\frac{1}{3}}]}^{3}$$

This paper describes the preparation of ceramic/polymer composite thick films via inkjet printing for dielectric applications. Therefore, BST is used as the ferroelectric ceramic component and PMMA as the polymeric component. Due to the use of a composite, no high temperature treatment is needed and the use of a flexible PET substrate is possible. In order to achieve a higher permittivity, two approaches are shown. On the one hand, the variation of the solids content in the composite film and on the other hand the increase of the ceramic particle size. The development of a highly versatile ink system allows the variation of the composition of the solids in the composite as well as the variation of the ceramic particle size. To investigate the dielectric properties of the printed composite thick films, fully inkjet printed metal insulator metal (MIM) capacitors are fabricated and characterized. Last, the experimental results are compared to the theoretical Eqs 2, 3, 4 and 5.

## Experimental Section

### Materials

BST was synthesized with a stoichiometric composition of Ba_0.6_Sr_0.4_TiO_3_: barium acetate (0.422 mol), strontium acetate hemihydrate (0.281 mol) and titanium isopropoxide (0.703 mol) were dissolved in acetic acid (30.0 mol) under nitrogen atmosphere. After the addition of water, the obtained sol was spray dried (MM-HT-ex laboratory spray dryer, Niro, Søborg, Denmark) and calcined. The precursor powders PPD1 and PPD2 were calcined under dried air for 1 h at 1100 °C and 1250 °C, respectively (CTF1600; Heraeus, Hanau, Germany). PMMA (*M*_w_ = 1.5 × 10^4^ g∙mol^−1^) was purchased from Sigma Aldrich and used without further modification.

### Ink preparation and characterization

First, phases of BST powder were characterized by XRD (Siemens D500 diffractometer, 40 kV, 40 mA) in a range of Bragg’s angle of 15° to 80° using Cu Kα (1.5406 A°) radiation with a step size of 0.04° at room temperature. Crystallite sizes were calculated with the Scherrer equation, by using a pseudo-Voigt profile to determine full width at half maximum of the peak. The composite inks were prepared by mixing of a BST dispersion with a PMMA solution. Therefore, the calcined BST powders PD1 and accordingly PD2 were milled and dispersed in butyldiglycol (BDG) using a laboratory stirred media mill (MiniCer; NETZSCH, Selb, Germany). BDG is used because of its high boiling point, high viscosity as well as its high surface tension. The solids content of the dispersions D1 and D2 was 39.4 wt% BST with an addition of 1.57 wt% KM3004 (Zschimmer & Schwarz, Lahnstein, Germany) as a dispersant. For PD1, calcined at 1100 °C, zirconia-grinding media *d* = 0.2 mm was used, while for PD2 the grinding media size was *d* = 0.4 mm. Samples of the dispersion were dried at 500 °C for SEM images. The particle size of the dispersions was determined using laser scattering (SLS; HORIBA LA950; Retsch Technology, Haan, Germany) and the solid content was determined using thermogravimetric analysis (STA 449 Jupiter, NETZSCH). The PMMA was dissolved in butanone, because of good solubility. A volume content of 20 or 15 vol% PMMA was used to ensure printability of the inks. BST dispersions were mixed with a PMMA solution to prepare three composite inks, with the desired volumetric ratio of BST to PMMA of 2:1 (inks A), 1:1 (inks B) and 1:2 (inks C). The calculated compositions of the used inks with dispersion BST-D1 (number 1) and BST-D2 (number 2) are displayed in Table 1.

**Table 1:** Compositions of the composite inks A, B and C by mixing of the displayed BST-dispersion and PMMA-solution.

Ink	*φ*_BST_ [vol%]	*φ*_PMMA_ [vol%]	*φ*_Disp_ [vol%]	*φ*_BDG_ [vol%]	*φ*_BUT_ [vol%]	BST dispersion [vol%]	PMMA solution [vol%]
A1	7.4	3.7	1.6	66.4	21.0	10	15
B1	6.6	6.6	1.4	59.1	26.3	10	20
C1	5.0	9.9	1.1	44.4	39.6	10	20
A2	6.9	3.5	1.5	68.5	19.6	10	15
B2	6.2	6.2	1.4	61.4	24.8	10	20
C2	4.7	9.5	1.1	46.7	38.0	10	20

For the characterization of the fluid mechanical properties the viscosities of the composite inks were measured using a rheometer (MCR 300; Anton Paar, Graz, Austria) with a cone-plate measurement geometry (*d*_cone_ = 25 mm, α_cone_ = 2°) and the surface tension was measured using a force tensiometer with the plate method (K100, Krüss, Hamburg, Germany). Densities were calculated.

### Inkjet printing

Inkjet Printing was performed with a single nozzle DoD inkjet printer (Autodrop Professional, Microdrop, Norderstedt, Germany). For both the composite inks and the silver inks, a 100 µm nozzle printhead was used. The distance between the nozzle and the substrate was set to 2 mm. All composite inks showed stable droplet formation around a driving voltage of *U*_Head_ = 90 V with a pulse length of *t*_Head_ ≈ 30 µs and an ejection frequency of *f* = 500 Hz. To obtain constant printing conditions the nozzle temperature was set to 25 °C and the substrate table was heated to 60 °C. Melinex® ST506™ (*d* = 125 µm, DuPont) was used as a flexible PET substrate. Both electrodes were printed using commercial silver nanoparticle inks. Silverjet DGP 40LT-15C was used for the bottom electrodes and SunTronic EMD 5730 for the top electrodes, because Silverjet ink showed a weak conductivity on top of a BST/PMMA layer. An optical microscope VHX-500FD (Keyence, Neu-Isenburg, Germany) was used for images of printed structures. Topography images were made using a chromatic white light interferometer (MicroProf; Fries Research & Technology, Bergisch Gladbach, Germany). Cross sections were prepared with an ion beam slope cutter (Leica EM TIC 3X, Leica Microsystems, Germany). Microstructures were investigated by SEM (Supra 55, Carl Zeiss, Oberkochen, Germany).

### Dielectric properties

The dielectric properties were characterized using an impedance analyzer (4294 A, Keysight Technologies, U. S.). Impedance *Z* and phase angle *δ* were measured with *U* = 1 V over a frequency range of *f* = 100 Hz – 200 kHz at room temperature.

## Results and Discussion

### Ink preparation

**Figure 1:**
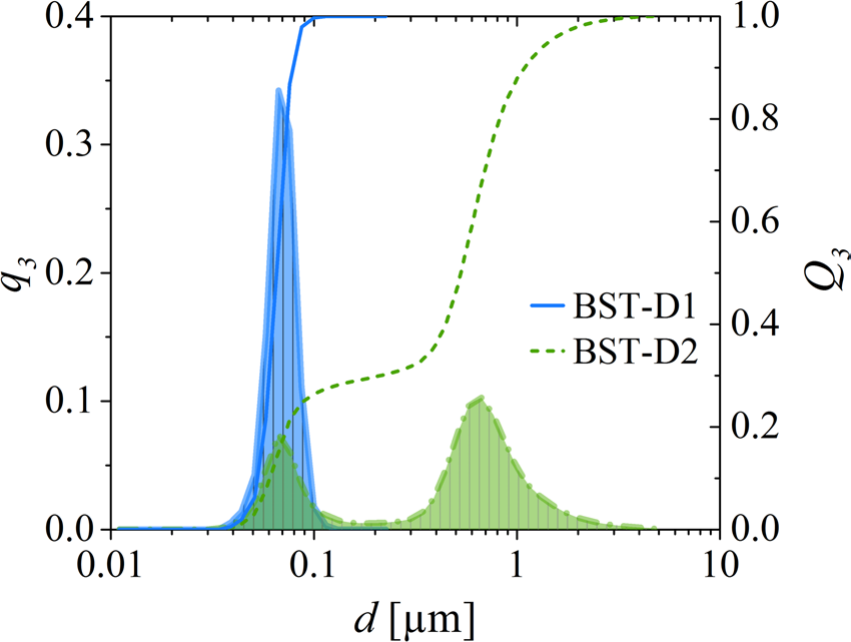
Particle size distribution measured by SLS of the BST dispersions BST-D1 and BST-D2.

**Figure 2:**
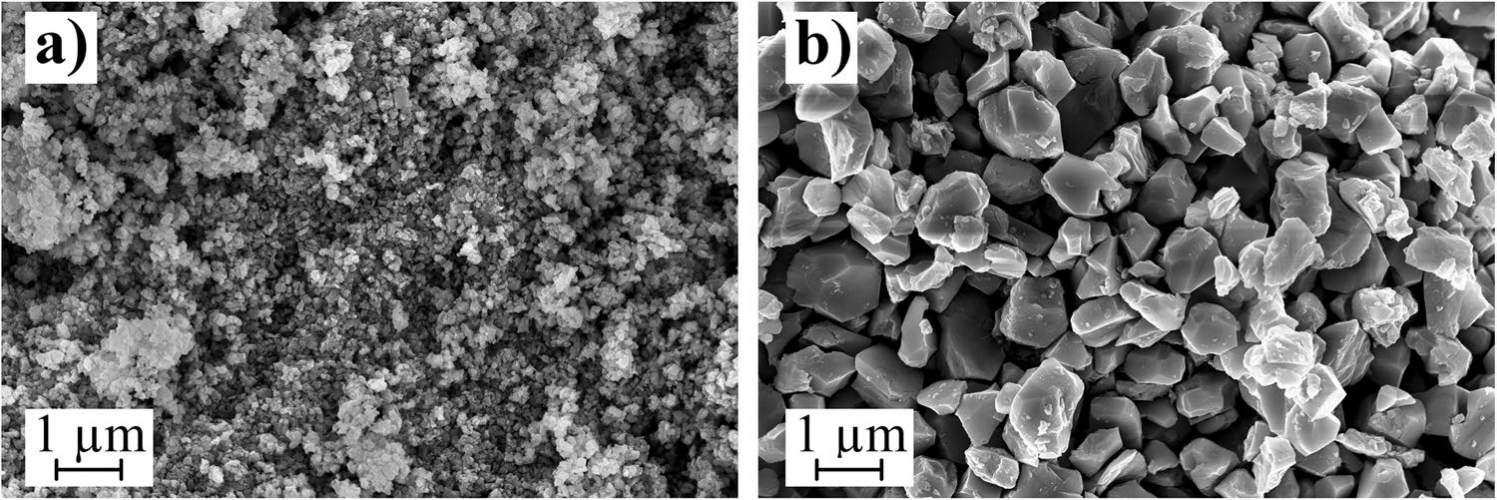
SEM images of BST particles after drying of the dispersions at 500 °C. (**a**) BST-D1 and (**b**) BST-D2.

In this section, the focus will be on the printability, the composition and the microstructure of the composites and the influence of the particle size on the dielectric properties. Therefore, two BST dispersions, BST-D1 and BST-D2, with a target BST content of 10 vol%, using butyldiglycol (BDG) as the solvent, are prepared. The preparation with an agitator bead mill was performed with different parameters and two differently calcined BST powders to obtain significant changes in the particle size distribution of the dispersions. The precursor of BST-D1 was calcined for 1 h at 1100 °C and of BST-D2 for 1 h at 1250 °C. The phase composition of both powders was confirmed via XRD and no foreign phases were detected (Fig. S1). Crystallite sizes are 697,7 Å (@ 2θ = 45,83°) for BST-D1 and 984,7 Å (@ 2θ = 45,78°) for BST-D2, with a lattice constant of a = 3,961 Å for both powders. The particle size distributions of the two dispersions are displayed in Fig. 1. While BST-D1 only contains particles in the small range of 60–130 nm (*d*_50,vol._ = 65 nm, *d*_90,vol._ = 79 nm), BST- D2 shows a bimodal distribution with ~^1^/_3_ volume percent of the particles in the range of 40–200 nm and ~^2^/_3_ volume percent in the range of 200 nm to 4 µm (*d*_50,vol._ = 0.53 µm, *d*_90,vol._ = 1.1 µm). Both the ratio of the particle sizes to one another and the composition of one third to two thirds is, according to various authors, well suited to achieve high packing densities of the particles^45–47^. To examine the results, SEM images of dried samples were taken, displayed in Fig. 2. Surface areas of the calcined powders are A_BST1100_ = 3,77 m²/g and A_BST1250_ = 0,77 m²/g (BET-method).

**Figure 3:**
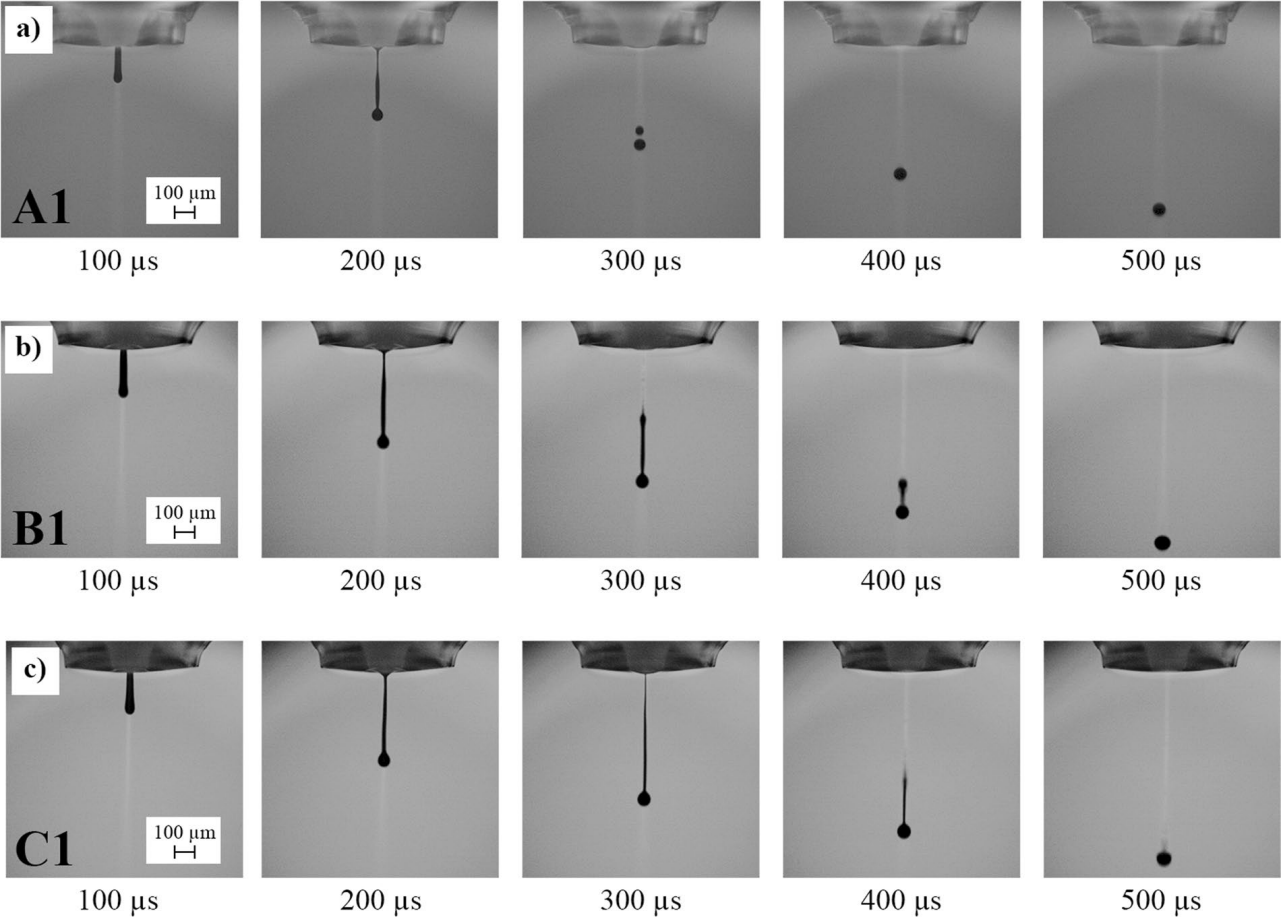
Time depending droplet formation of the inks A1, B1 and C1.

To evaluate the influence of the composite composition on the dielectric properties, three different ratios of BST and PMMA in the composites were investigated. Dried structures are composed of *φ*_BST_ = 0.67 and *φ*_PMMA_ = 0.33 (inks A1&2), *φ*_BST_ = 0.5 and *φ*_PMMA_ = 0.5 (inks B1&2) and *φ*_BST_ = 0.33 and *φ*_PMMA_ = 0.67 (inks C1&2). The composite inks A1 to C1 were prepared by addition of the PMMA solutions to dispersion BST-D1. The inks A2 to C2 were prepared with BST-D2. The compositions between the inks with number 1 and 2 differ slightly (Table 1), since BST-D2 only had a BST volume content of 9 instead of 10 vol%. The lower fraction of solids is due to a stronger sedimentation of the larger ceramic particles in the mill or pipes during the grinding process. Hence, also the fluid mechanical properties shown in Table 2 differ slightly. Nonetheless, the inks show nearly the same rheological behavior (Fig. S2**)** inks are suitable for printing and show a stable droplet formation as expected due to the values for *Oh*^48^. The droplet formation for the three inks is displayed in Fig. 3. The forming filament between the nozzle and the droplet of the ink C1 remains longer stable than for the inks A1 and B1. This is due to the number of polymer chains and their orientation in the direction of flow through the capillary. As a result of the interactions, the filament can remain stable for a longer time until it detaches from the nozzle^49,50^. However, the printing paramaters were adjusted to nearly equal droplet velocities in flight of about *v*_droplet_ = 2 m/s for all inks.

**Table 2:** Fluid mechanical properties of the composite inks A, B and C1 (BST-D1) and A, B and C2 (BST-D2).

	*ρ* [g/cm³]	*η* [mPa∙s]^†‡^	*σ* [mN/m]^†^	*Oh* ^*1*^
A1	1.28	7.5	29.6	0.12
B1	1.24	8.6	29.1	0.14
C1	1.15	8.8	27.0	0.16
A2	1.27	6.6	30.5	0.11
B2	1.23	7.5	30.1	0.12
C2	1.15	6.7	29.7	0.11

^†^*T* = 20 °C, ^‡^*γ*’ = 1000 s^−1^, ^1^*d*_head_ = 100 µm.

### Capacitor fabrication

**Figure 4:**
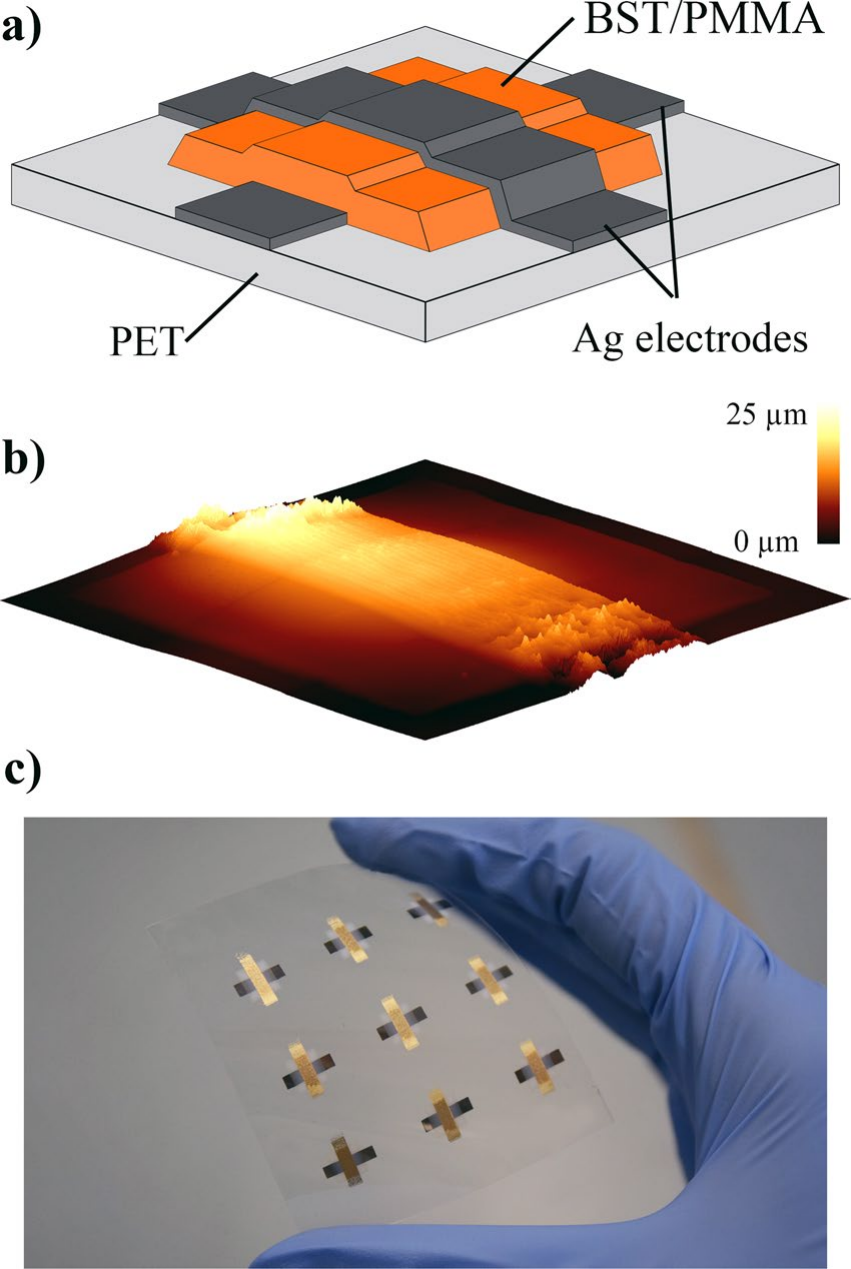
(**a**) Schematic layout for the printed capacitors; (**b**) Topography of a fully printed capacitor with ink A1; (**c**) Picture of a PET substrate with nine printed samples.

The schematic layout of the fully printed MIM capacitors is shown in Fig. 4a. First, the bottom electrode is printed as a single layer and dried for 30 min on the printing table. The composite thick film is then printed on the subsequent day. A single layer BST/PMMA thick film results in a more homogeneous topography and less failures compared to double printed films^33,51^. After a further drying period of 30 min on the printing table, the top electrode is printed with two layers to get a consistently layer on the composite. Figure 4b shows the topography of a fully printed capacitor with ink A1. As it can be seen, the topography of the composite layer is very homogeneous without any irregularities. Furthermore, the top electrode shows a good quality on the dielectric layer, but the transition area on the PET shows an uneven surface due to the differences in the drying behavior on the PET and on the composite layer. Hence, the top electrodes were electrically contacted on the edges of the composite layers instead of the PET film. In Fig. 4c, a standard substrate with nine printed capacitors is shown. For the dielectric characterizations, at least a minimum of two substrates for each ink were fabricated.

Because of the use of two silver inks with very different manufacturer data concerning the after treatment, the conductivity of the printed electrodes was examined as a function of temperature treatment. The samples were treated in the oven at 80 °C, 100 °C, 120 °C and 140 °C for 1 hour. According to the manufacturer, the used PET substrate is stable up to a temperature of 160 °C. However, the substrates showed a clear discoloration and became cloudy even at 140 °C.

**Figure 5:**
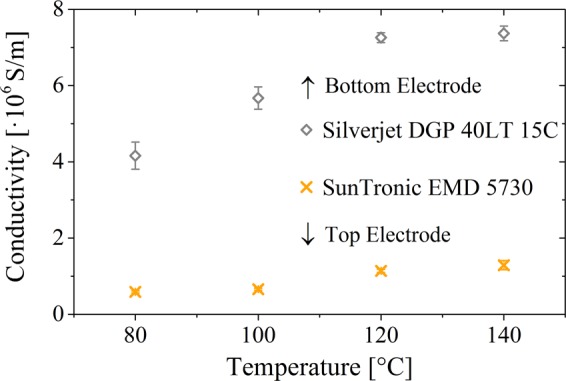
Conductivity of the bottom and top electrodes treated for 1 h at different temperatures and characterized via 4-wire sensing.

The conductivity was characterized using a special layout for 4-wire measurements (Fig. S3). Therefore, the bottom electrode was printed on PET and the top electrode was printed on a large composite film of ink B1 with a composite ratio of 1:1. The results are displayed in Fig. 5. A temperature treatment of 120 °C for 1 hour presents the best results for the conductivity, while still being a gently treatment for the PET as well as the capacitors. With this treatment, the bottom electrode shows a specific resistivity of 13–14 µΩ·cm (According to manufacturer: 11 µΩ·cm) and the top electrode of 85–90 µΩ·cm (According to manufacturer: 5–30 µΩ·cm).

**Figure 6:**
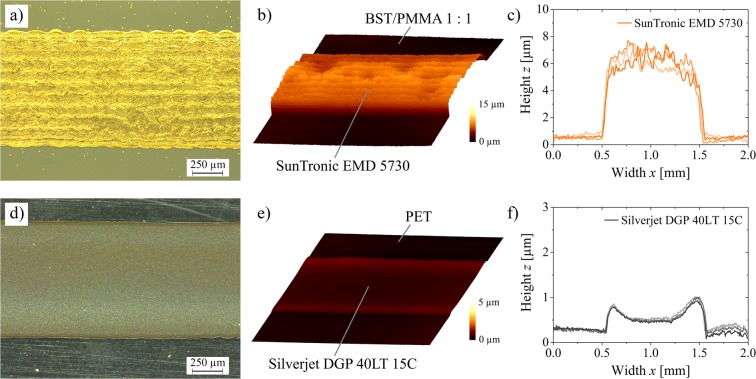
Overview of the printed electrodes after temperature treatment of 120 °C for 1 h. Upper row (**a–c**) displays the top electrode and lower row (**d–f**) the bottom electrode. Optical microscope images (**a,d**), topography images (**b,e**) and 2D-profiles (**c,f**).

The structure and quality of the electrodes used for conductivity measurements are shown in Fig. 6. The same results are obtained for the electrodes in printed capacitors. The individual drops form dense and homogeneous layers in both electrodes. However, the two-layer top electrode clearly shows residues of the individually printed lines in its topography. The surface is relatively rough and exhibits significant variations, but this is negligible for the manufacturing of the capacitors. The surface of the lower electrode shows an edge elevation due to a slight coffee stain effect, but at the same time is uniform without the occurrence of spikes. The height of the lower electrode is in the range of 300 nm in the middle up to about 800 nm at the edges. The upper electrode has a thickness of 4.5–6.5 μm. Overall, sufficiently good electrodes for the production of fully printed capacitors could be fabricated.

### Microstructure and morphology

**Figure 7:**
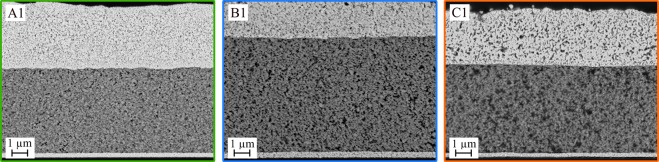
Cross sectional SEM images of fully printed capacitors based on BST dispersion D1; (**a**) Ink A1 (67 vol% BST); (**b)** Ink B1 (50 vol% BST); (**c**) Ink C1 (33 vol% BST).

The microstructure of the printed BST/PMMA layers was examined by ion-etched cross-sections, prepared after the temperature treatment of 120 °C for 1 h and the dielectric characterization. This method avoids a direct mechanical load, whereby the porosity is also maintained with materials of different hardness. Figure 7 shows SEM images of capacitors fabricated with the three different composite inks A1, B1 and C1. All three composite films show a very homogeneous microstructure and distribution of the particles. BST and PMMA are expected to show good physical interactions and can form a network-like structure during drying^52–54^. Therefore, no clustering of the BST or PMMA was observed and only a very small porosity was identified by optical analysis.

Despite the temperature treatment of the printed capacitors of 120 °C for 1 h, which is above the glass transition temperature of PMMA, no significant porosity formation occurs by micro Brownian motions of the PMMA chains. The B1 and C1 films have a negligible porosity of 0.2% and <0.1%, respectively. The A1 film with the highest BST content shows a slightly higher porosity of 1.2%, since there is less PMMA between the ceramic particles. As before, the SEM images show a uniform surface for the lower electrode as well as for the three composite compositions. In addition, the transition regions are clearly separated from one another and no intermixing was observed during the printing and drying of the different layers. This is especially important for the printing of the upper electrode since penetration of the silver particles can lead to short circuits.

**Figure 8:**
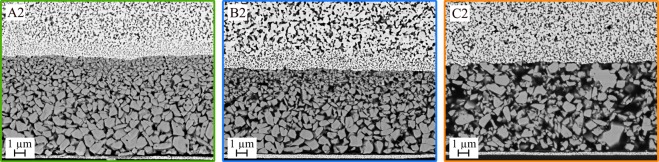
Cross sectional SEM images of fully printed capacitors based on BST dispersion D2; (**a**) Ink A2 (67 vol% BST); (**b**) Ink B2 (50 vol% BST); (**c**) Ink C2 (33 vol% BST).

In comparison to the very homogeneous films shown in Fig. 7, the microstructures of the composite films printed with dispersion BST- D2 show large differences. Three cross sectional images of capacitors are illustrated in Fig. 8. First, the size of the ceramic particles is highly increased and therefore the particles are clearly visible. However, the large particle size leads to significant sedimentation of the particles during the time of drying, especially in the inks with higher BST volume content. While the particles are embedded homogeneous in the PMMA matrix using ink C2, the films of ink A2 and B2 show a clear separation of the larger and the smaller particles in the dried layers. Ink B2 still contains enough PMMA to result in a very dense microstructure without porosity (<0.5%), whereas the composite film of A2 shows a porosity of about ~11%. The prevention of sedimentation in ink C2 can be well explained by the increase of the viscosity during drying as well as by the formation of a network-like structure, up to a gel-character. A detailed study of the drying behavior of our developed BST/PMMA inks has already been reported^55^. Decisive for homogeneous structures is the formation of a physical network between the particles and the polymer chains. This network becomes stronger with increasing PMMA content. Ink C2, with the highest PMMA content shows a gel-like character even with the larger BST particles and hence, the sedimentation is hindered. The viscosities of A2, B2 and C2 after evaporation of BUT compared to A1, B1 and C1 are shown in Fig. S4. Evaporation of BUT occurs very fast and the BUT content is negligible for the drying behavior. In addition, oscillation measurements of C1 and C2 are displayed in Fig. S5, which confirm the gel-character. With regard to the obtained microstructure, it can be stated that the inks containing only the small BST particles of dispersion BST-D1 are more favorable.

### Dielectric characterization

**Figure 9:**
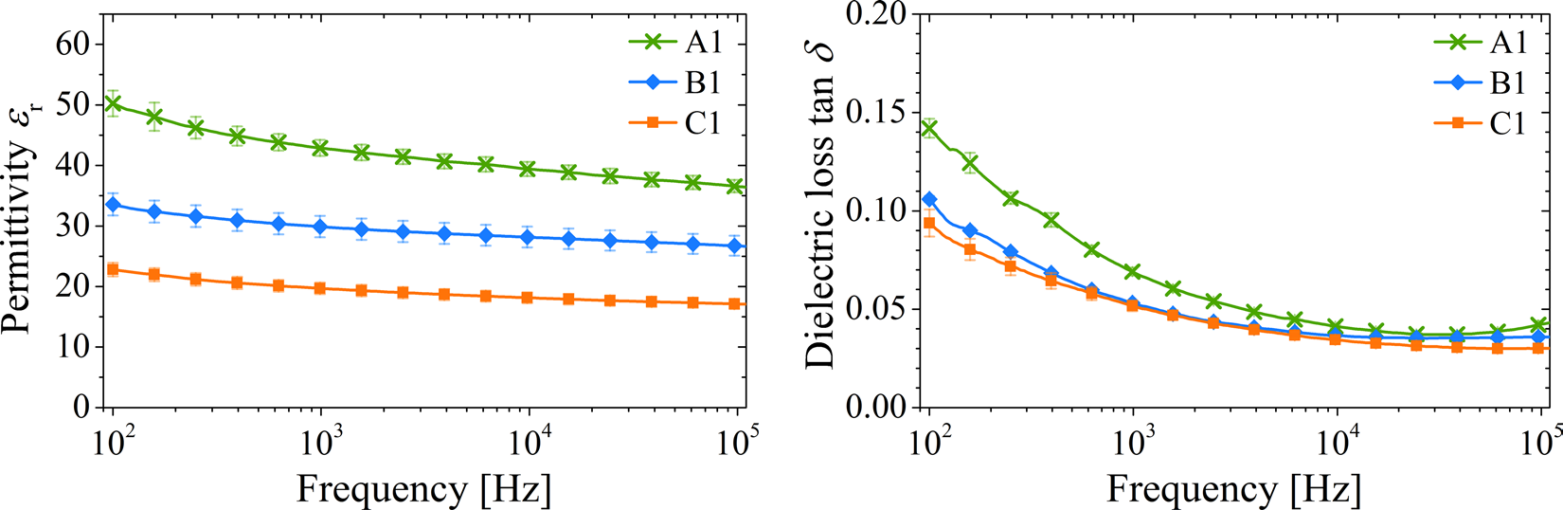
Dielectric properties of the dielectric thick films prepared with (A1) (66 vol% BST), (B1) (50 vol% BST) and (C1) (33 vol% BST). Left side: dielectric constant; right side: loss tangent.

The permittivity *ε* and the dielectric loss tan *δ* of the printed BST/PMMA composite films were characterized over a frequency range of 100 Hz to 200 kHz. The values for the impedance *Z* and the phase angle *φ* were measured and *R* and *χ* were calculated. Layer thicknesses were determined via SEM-images. Based on that, the capacitance *C*, the permittivity *ε*_r_ and the loss tangent tan *δ* were determined^56^. The results for A1 (*φ*_BST_ = 0.67), B1 (*φ*_BST_ = 0.50) and C1 (*φ*_BST_ = 0.33) are shown in Fig. 9.

All three composites show promising results for the permittivity in comparison to reported PMMA/BT composites fabricated by different methods^52,53,57–59^. Despite the very small particle size and the processing in the form of printed inks, comparable and higher values for the permittivity are achieved. This increases significantly with increasing BST filler content. At the same time, an increase in frequency causes a slight decrease in permittivity. The printed composite layers thus show the behavior of the individual materials and are consistent with comparable investigations of other ceramic/PMMA composites^60,61^. Comparing the values for *ε*_r_ at *f* = 1 kHz of the different layers A1 (~42), B1 (~30) and C1 (~20) with those of pure PMMA (~3)^62^, an increase in the permittivity by the factor 7–14 was achieved with the printed composite films. At low frequencies, higher values are obtained for the loss factor due to movement within the polymer chains and interfacial polarization^63,64^.

**Figure 10:**
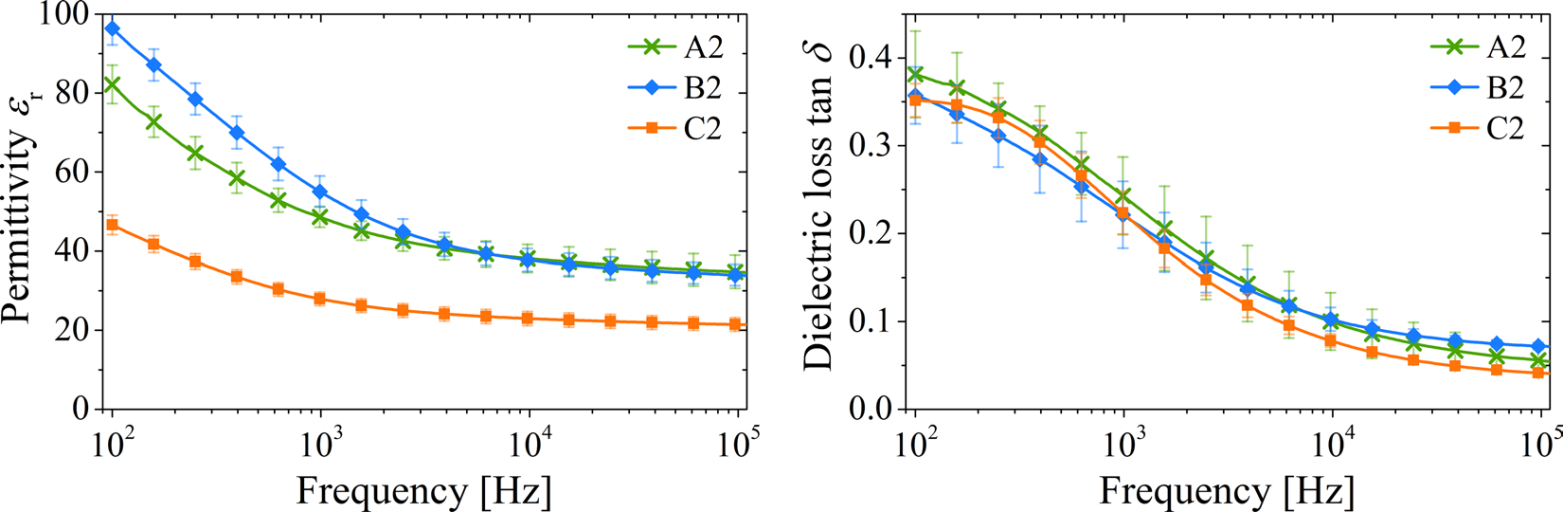
Dielectric properties of the dielectric thick films prepared with (A2) (66 vol% BST), (B2) (50 vol% BST) and (C2) (33 vol% BST). Left side: permittivity *ε*_r_, right side: loss tangent tan *δ*.

The results of the frequency-dependent dielectric characterization of the three composites containing the larger BST particles are shown in Fig. 10. For frequencies less than 1 kHz, the layers exhibit a strong increase in permittivity compared to the layers with BST-D1. This effect was intended by the use of a larger particle size, which is already described by different authors^30,37,65^. At the same time, the layers with larger particles show a much higher loss factor tan *δ*, which assumes comparable values to the A–C1 capacitors only in the range *f* = 100 kHz. The phase shift differs more strongly from the ideal −90° when the BST-D2 dispersion is used. The negative imaginary part *χ* of the impedance shows no significant differences for the different samples. The resistance *R* increases in comparison with the samples with BST-D1. Since the manufacturing process – including electrode fabrication as well as the contacting of the electrodes – was identical for all samples, manufacturing-induced influences should be negligible. The higher losses are therefore mainly attributable to losses in the dielectric layer. The field forces which arise during the polarity reversal of the capacitor plates act on the electron shells and dipole structures in the dielectric^66^. These can be adversely affected by the greater inhomogeneity caused by the broad particle size distribution. In addition, there is the possibility that the BST-D2 composite layers have a higher conductivity and thus result in higher losses^67^.

Contrary to the expectation from the experiments with BST-D1, the B2 composite shows a higher permittivity than the A2 film up to 6 kHz, although the BST content is lower. At higher frequencies, both compositions have nearly identical values for *ε*_r_. For a detailed understanding of these results, the consideration of the microstructure shown in Fig. 8 is essential. Due to the sedimentation of the larger particles, the printed layer should not be considered as a uniform layer. Rather, the printed film can be considered as two stacked layers. The composition of the A2 microstructure was characterized on the assumption that the small particles with *d* ≤ 200 nm form the upper layer and the larger particles the bottom layer. The proportion of the small particles in the BST-D2 dispersion is about 30% by volume (Fig. 1). If the desired composition of BST and PMMA of *φ*_BST_ = 0.67 and *φ*_PMMA_ = 0.33 is achieved for the small particles, also 30% by volume of the present PMMA is bound to the small BST particles. In addition, the high porosity of the A2 film (11%) has to be taken into account and must be included with a factor of 0.3. This results in the A2 microstructure consisting of two layers, of which the upper layer represents 33% of the composite thickness.

The compositions of the complete A2 film as well as of the two separate layers are shown in Table 3. First, it can be seen that the striven composition between BST and PMMA of 2:1 is achieved in the film. However, due to the high porosity the overall composition is different to the pores, which have a dielectric constant of one and should be considered as a part of the low dielectric component. Hence, the division into two layers is necessary. The upper 33% overall only contain slightly more than 50 vol% BST, while the bottom 67% layer consists of around 60 vol% BST. Therefore, the dielectric properties will be different between the upper and the lower layer, especially considering that the particle size is different, too. Consequently, Eq. (1) must be used to describe the effective permittivity of the A2 film and it becomes clear that the layer structure reduces the effective permittivity, as it was determined experimentally in Fig. 9.

**Table 3:** Composition of the printed A2 composite film and the division into two layers.

	Complete [vol%]	Top 33% [vol%]	Bot 67% [vol%]
BST	58.0	51.5	61.3
Organic	30.4	38.5	26.4
Pores	11.6	10.0	12.3

**Figure 11:**
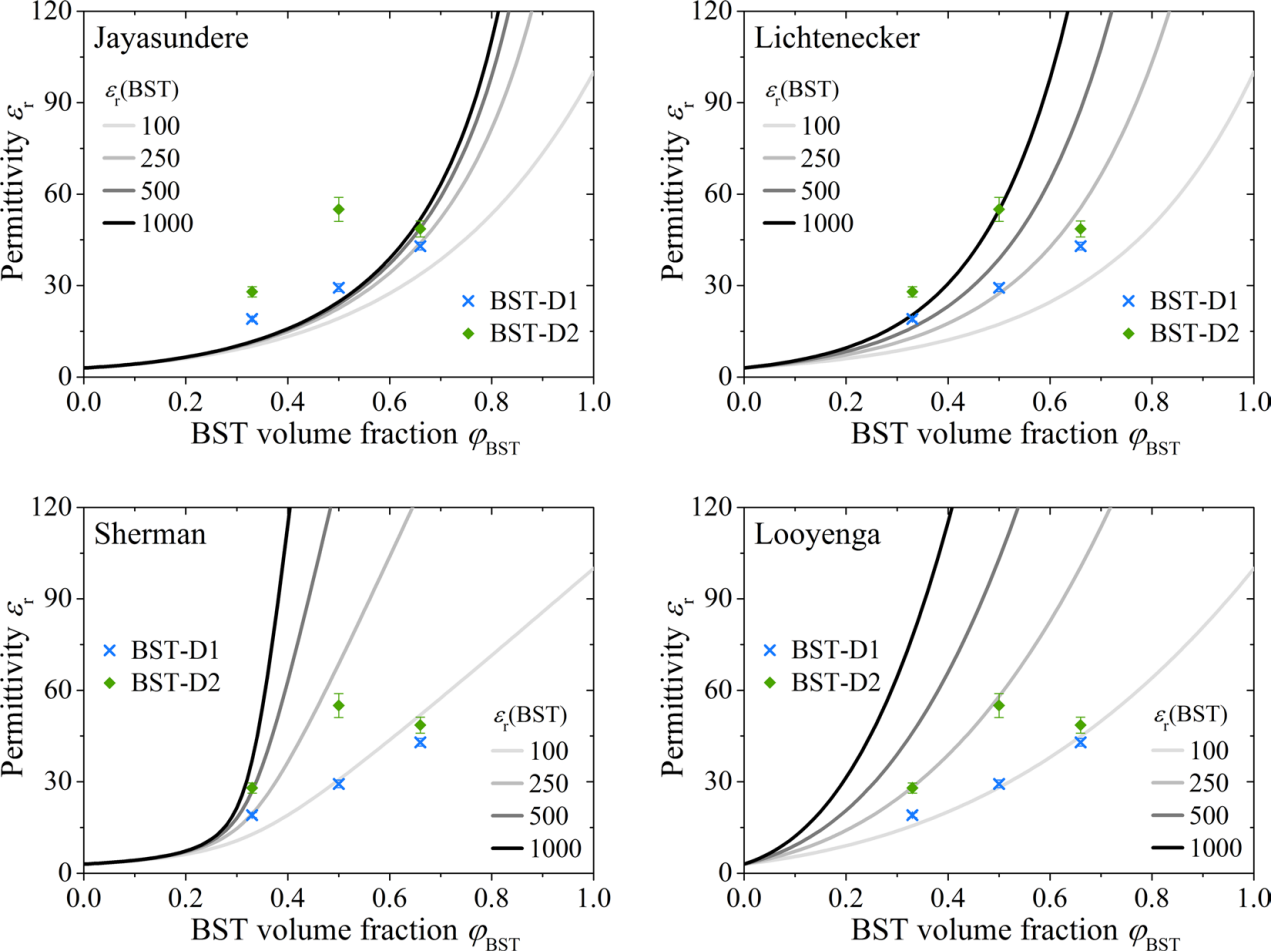
Comparison of the predictions by Jayasundere, Lichtenecker, Sherman and Looyenga equation calculated with different values for *ε*_r_ (BST powder) and the experimental values for the printed BST/PMMA compositions at *f* = 1 kHz.

The experimental results for the dielectric constant are compared to the described theoretical models of Jayasundere, Lichtenecker, Sherman and Looyenga. The experimental values for *ε*_r_ of the composite layers at a frequency of *f* = 1 kHz with both BST-D1 and BST-D2 are shown together with the calculated theoretical curves in Fig. 11. The theoretical flow curves were calculated with *ε*_r_ = 3 for PMMA and with *ε*_r_ = 100, 250, 500 and 1000 for the initial BST powder.

The theoretical models differ significantly in their curve progression and consistency with the experimental results. The model according to Jayasundere delivers significantly lower values than measured up to a BST volume content of *φ*_BST_ = 0.5 even with an initial permittivity for the BST powder of *ε*_r_ = 1000. The model is strongly based on the interactions between the particles of the filler, which is why the curves increase steeply at higher filling levels. The model according to Lichtenecker also shows no good agreement with the measured values. The experimental results cannot be attributed to any particular initial permittivity value of the BST powder. However, the model is often used in the literature with good conformity^30,37,68^. Like the Jayasundere model, also the Lichtenecker equation provides too high values for the initial BST permittivity in our printed composites when doing a back calculation. Considering the small particle size and the abstinence of sintered necks, these values are unrealistic and the two models are not suitable for the investigated system^37,69^.

The Sherman equation, which is based on the considerations of Bruggeman, first provides an acceptable agreement with the experimental data for the BST-D1 composite layers. The assumed permittivity of the BST-D1 starting powder appears to be in a realistic range of about 100. However, this model is not adequately suited for a good predictability of the properties of the different BST/PMMA compositions, especially in the range of lower BST contents and for the larger BST particles.

By far the best agreement is obtained with the Looyenga model, which is also based on the model of Bruggeman. The experimental values for the BST-D1 composite layers show an excellent agreement with the model using the assumption for the starting powder of *ε*_r_ (BST-D1 powder) = 100. Furthermore, the values of the BST-D2 layers are in good agreement with the assumption *ε*_r_ (BST-D2 powder) = 250. Only the measured value for the A2 composite is substantially different. However, this is not surprising with regard to the previously discussed microstructure (Fig. 8), since both the high porosity as well as the formation of a layered structure inside the composite film are strong special effects.

Taking into account the layer structure and the resulting series circuit, the model according to Looyenga can also provide a good match for the experimental A2 value. The lower 67% layer of A2 effectively has a BST fill level of *φ*_BST_ = 0.61. Looyenga provides a calculated value of the composite of *ε*_r_ = 85 with *ε*_r_ (BST) = 250. The upper 33% layer has a BST content of *φ*_BST_ = 0.51 and a particle size comparable to the BST-D1 dispersion. Therefore, the measured value for B1 (*ε*_r_ = 30) can be used for the upper layer while neglecting the porosity. Using Eq. (1) for the printed A2 film and the percentage distribution of the layers described above, a computational value for the permittivity of the printed A2 film of 53 is obtained. This theoretical value corresponds almost to the experimentally determined value of *ε*_r_ (A2) = 49 ± 3. Considering a slight reduction of the permittivity of the non-ceramic components (PMMA + pores) by *ε*_r_ (air) = 1, an even better match should be achieved.

Based on the models discussed, the Looyenga equation is very suitable for the printed BST/PMMA composites. A good prediction of the dielectric properties, especially with the use of the dispersion BST-D1, is given here.

## Conclusions

The successful fabrication of fully inkjet printed MIM capacitors based on BST/PMMA composite inks on flexible substrates was demonstrated. Therefore, three composite inks were developed with different ratios of BST to PMMA. Dried structures are composed of *φ*_BST_ = 0.67 and *φ*_PMMA_ = 0.33 (inks A), *φ*_BST_ = 0.5 and *φ*_PMMA_ = 0.5 (inks B) and *φ*_BST_ = 0.33 and *φ*_PMMA_ = 0.67 (inks C). The simple preparation route of the composite inks allows a one-step fabrication of the dielectric films combined with a high reproducibility. The structural characterization of the printed films showed very homogeneous surfaces and particle distributions with smooth transitions between the different layers in the capacitors. No signs of blending between the used silver inks and the BST/PMMA inks were observed. The inks are highly suitable for the fabrication of multi-layer components.

For the investigation of the dielectric properties, two ceramic dispersions with different particle size distributions were prepared and used in each of the three composite inks. The inks with an unimodal particle distribution in the range of 40–130 nm showed values for the dielectric constant *ε*_r_ at 1 kHz of 20 (C1, 33 vol% BST), 30 (B1, 50 vol% BST) and 42 (A1, 67 vol% BST) for the composites. The films fabricated with the bimodal particle distribution with ~^1^/_3_ vol% of the particles in the range of 40–200 nm and ~^2^/_3_ vol% in the range of 300 nm to 4 µm showed an increase of the permittivity to 28 (C2, 33 vol% BST), 55 (B2, 50 vol% BST) and 49 (A2, 67 vol% BST). Due to the sedimentation of the larger BST particles in the printed films of A2 and B2 no further increase of the dielectric constant was obtained, because a serial capacitor circuit was formed in the printed microstructures. Nonetheless, the dielectric constant of the printed composites was about 7 to 18 times higher, compared to pure PMMA. The comparison to different theoretical models for the prediction of the dielectric properties of the different composite compositions showed particularly good agreement with the model of Looyenga. This allows a reliable prediction of the dielectric properties of the printed BST/PMMA composites. Our experimental study delivers reproducible results to fully inkjet printed ceramic/polymer composites containing MIM capacitors. The correlation of the printed microstructures with the dielectric properties was successfully demonstrated and we were able to show that inkjet printing of composite inks is a promising fabrication method for fully printed dielectric devices.

## Supplementary information

Supplementary Information

Dataset S1–S7
