# Supplementary material for: Fabrication and Characterization of Fully Inkjet Printed Capacitors Based on Ceramic/Polymer Composite Dielectrics on Flexible Substrates
Source: Sci Rep. 2019 Sep 16;9:13324. doi: 10.1038/s41598-019-49639-3 (PMC6746780; doi:10.1038/s41598-019-49639-3)
Supplement: Supplementary file 1 — Supplementary Information [file 41598_2019_49639_MOESM1_ESM.docx]

Fabrication and Characterization of Fully Inkjet Printed Capacitors Based on Ceramic/Polymer Composite Dielectrics on Flexible Substrates

Morten Mikolajek, Timo Reinheimer, Nicole Bohn, Christian Kohler, Michael J. Hoffmann, Joachim R. Binder*

Institute for Applied Materials, Karlsruhe Institute of Technology, Hermann-von-Helmholtz-Platz 1, 76344 Eggenstein-Leopoldshafen, Germany.

E-Mail address: Joachim.Binder@kit.edu

**Supplementary Information**

Fig. S1: XRD-spectra of Ba_0.6_Sr_0.4_TiO_3_

Fig. S2: Shear rate depending viscosities of the used composite inks

Fig. S3: Layout of the 4-wire conductivity measurements

Fig. S4: Shear rate dependency of the viscosity of the inks after evaporation of Butanone

Fig. S5: Oscillation measurements of the inks C1 and C2

Fig. S6: Image analysis of the porosity and ceramic content of films with BST-D1

Fig. S7: Image analysis of the porosity and ceramic content of films with BST-D2

**Fig. S1.** XRD-Spectra of Ba_0.6_Sr_0.4_TiO_3_ powders, calcined at 1100 and 1250 °C. No foreign phases were detected.


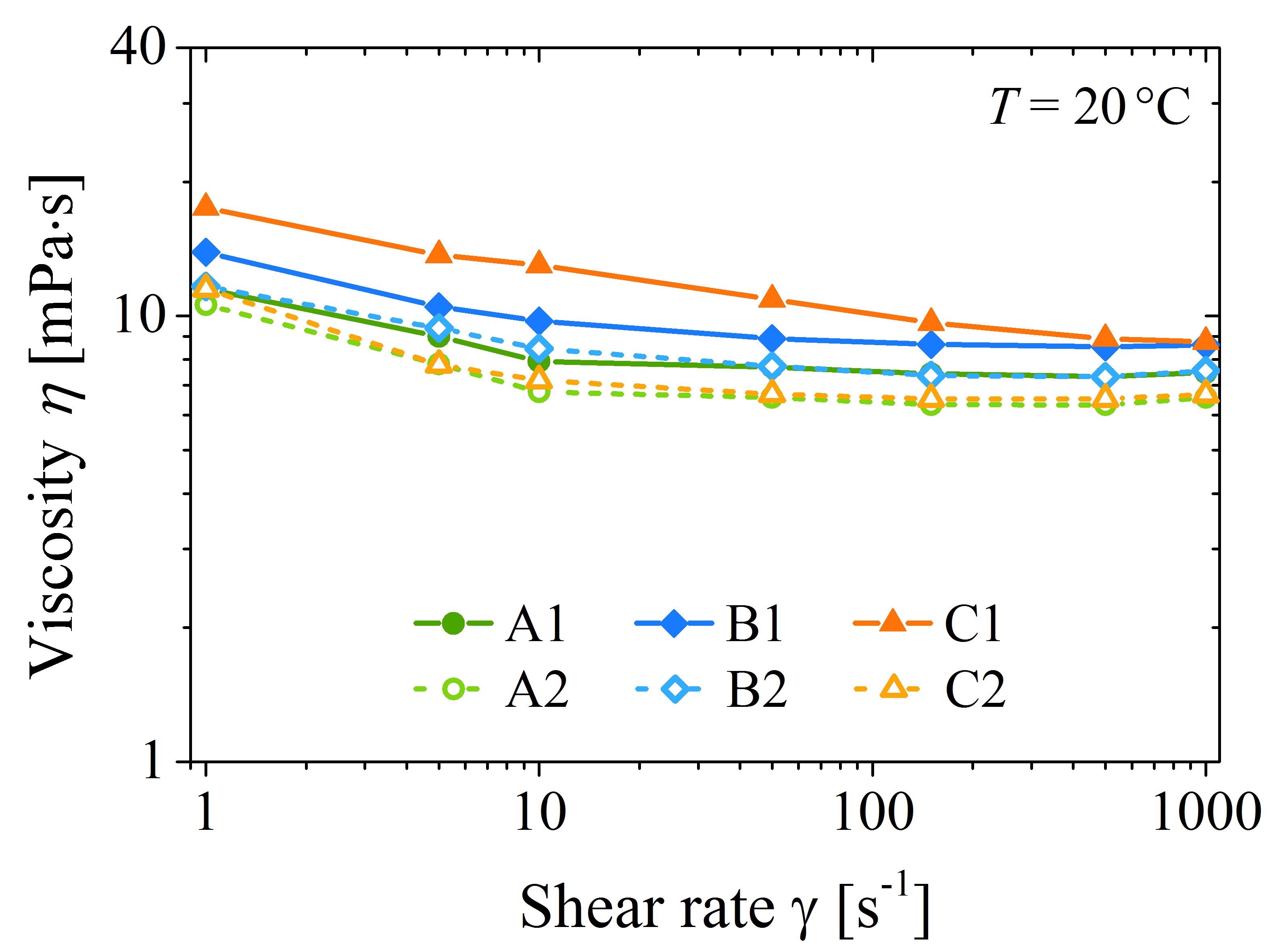


**Fig. S2.** Shear rate depending viscosities of the used composite inks in this study at *T* = 20 °C.


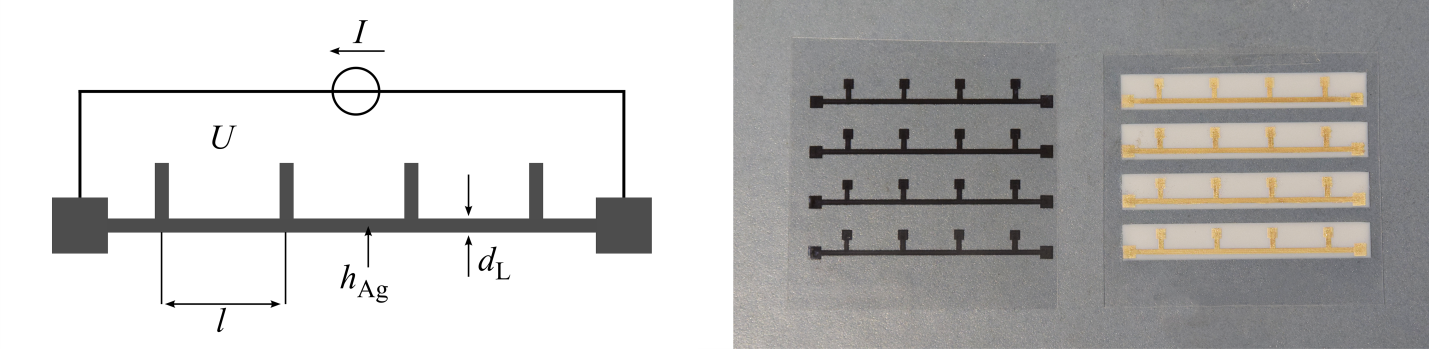


**Fig. S3.** Layout of the 4-wire conductivity measurements (left) and printed examples (right)


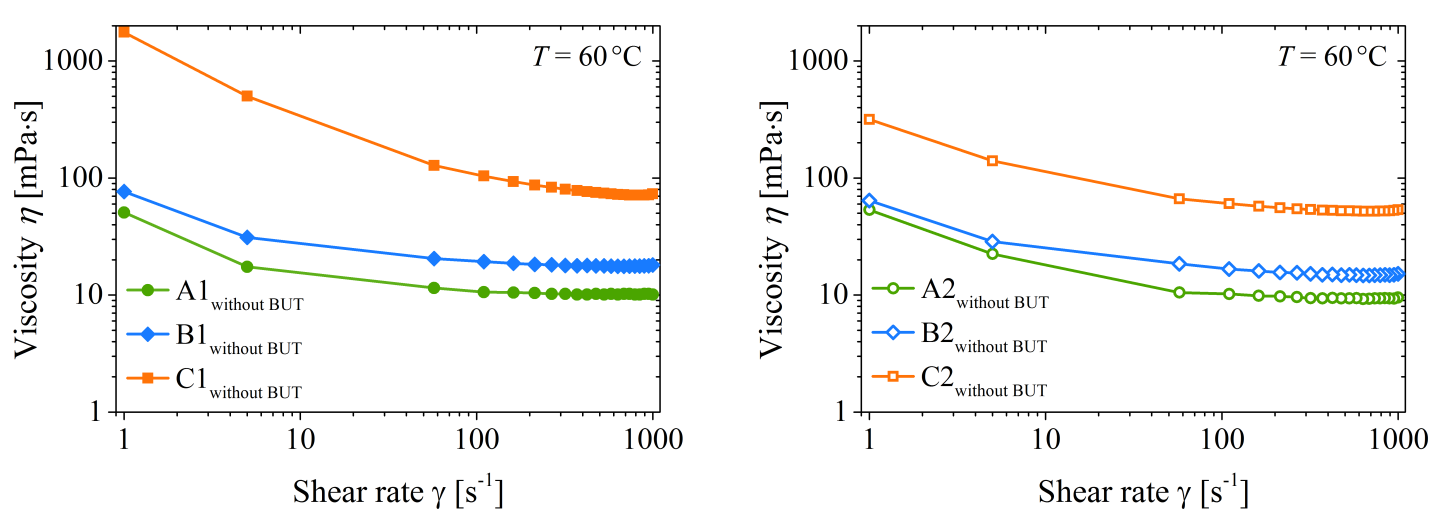


**Fig. S4.** Shear rate dependency of the viscosity of the inks with BST-D1 (left) and BST-D2 (right) at 60 °C after the butanone content was removed with a rotary evaporator.


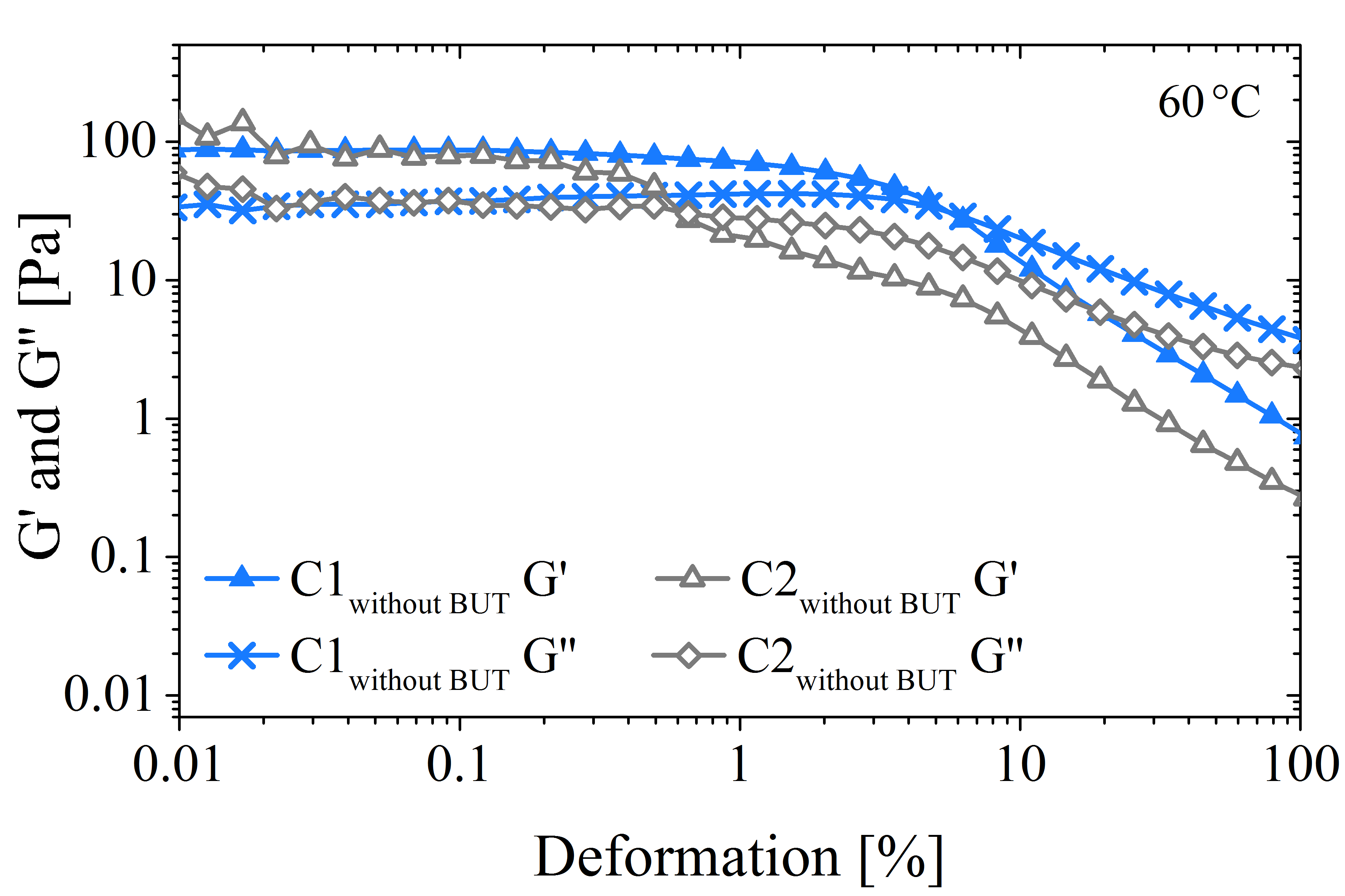


**Fig. S5.** Oscillation measurements of the inks C1 and C2 after evaporation of butanone.


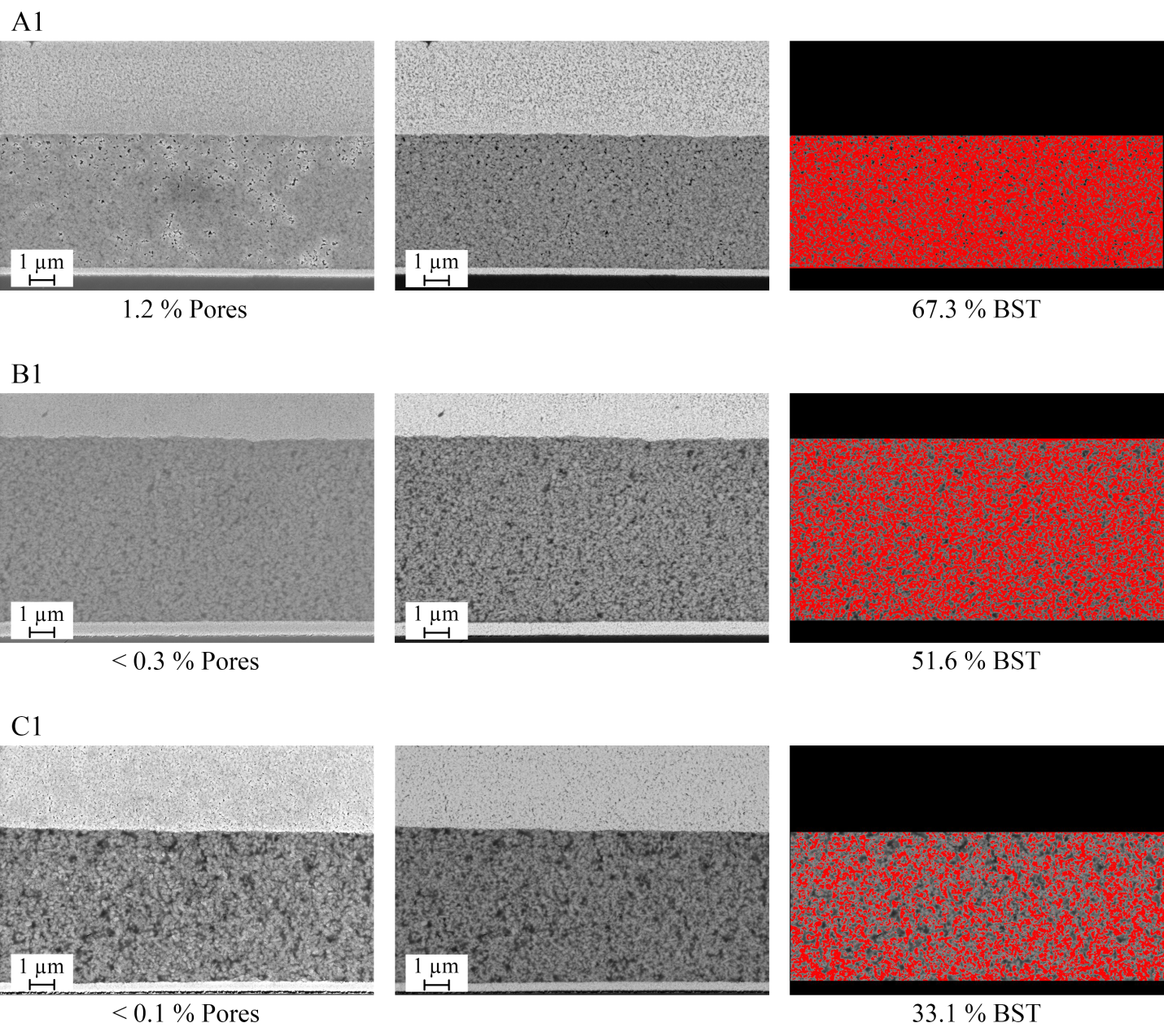


**Fig. S6.** Image analysis of the porosity and ceramic content of the printed films with BST-D1 using SEM cross sections.


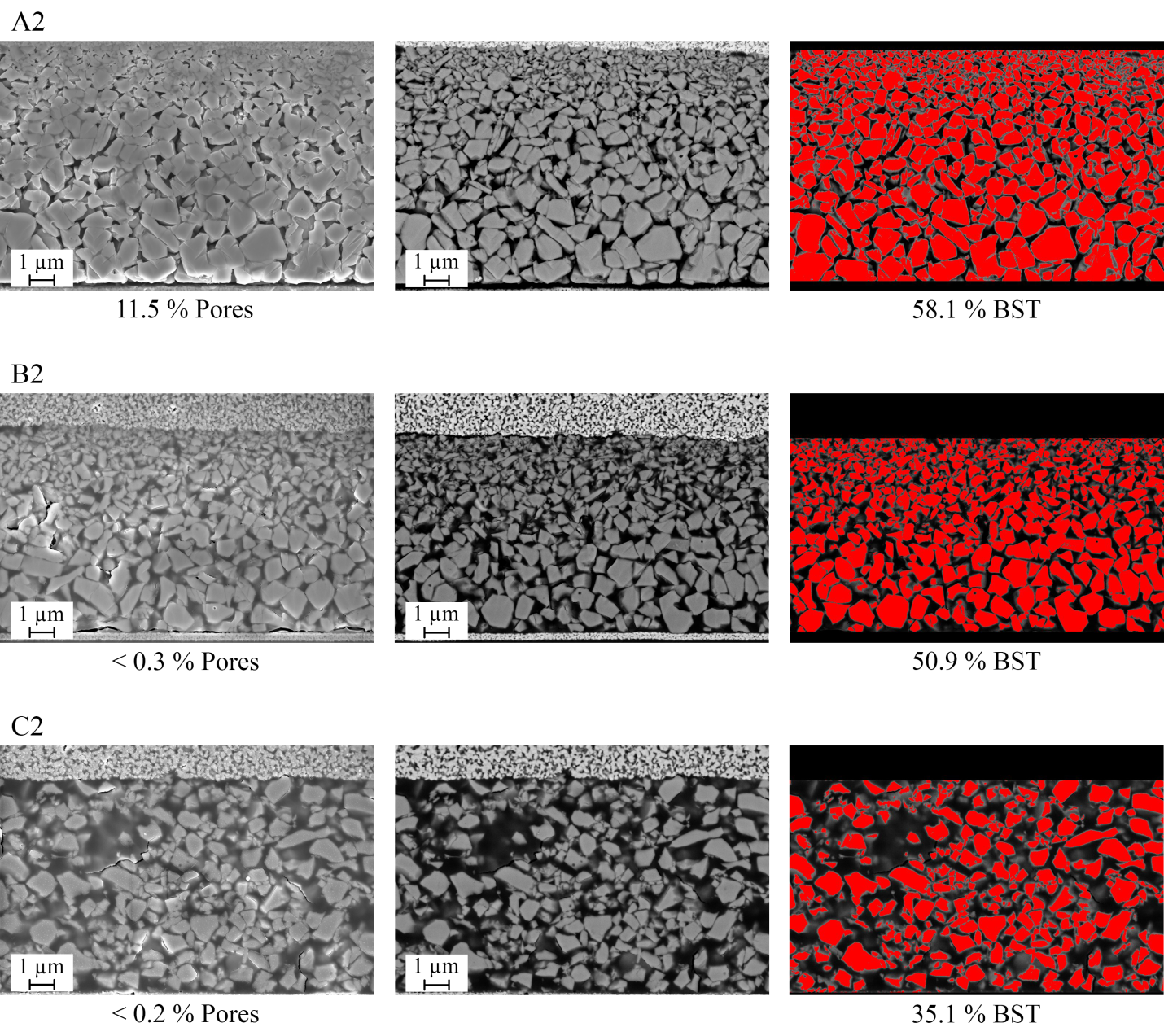


**Fig. S7.** Image analysis of the porosity and ceramic content of the printed films with BST-D2 using SEM cross sections.
