# Supplementary figures and images for: Fabrication and Characterization of Fully Inkjet Printed Capacitors Based on Ceramic/Polymer Composite Dielectrics on Flexible Substrates
Source: Sci Rep. 2019 Sep 16;9:13324. doi: 10.1038/s41598-019-49639-3 (PMC6746780; doi:10.1038/s41598-019-49639-3)

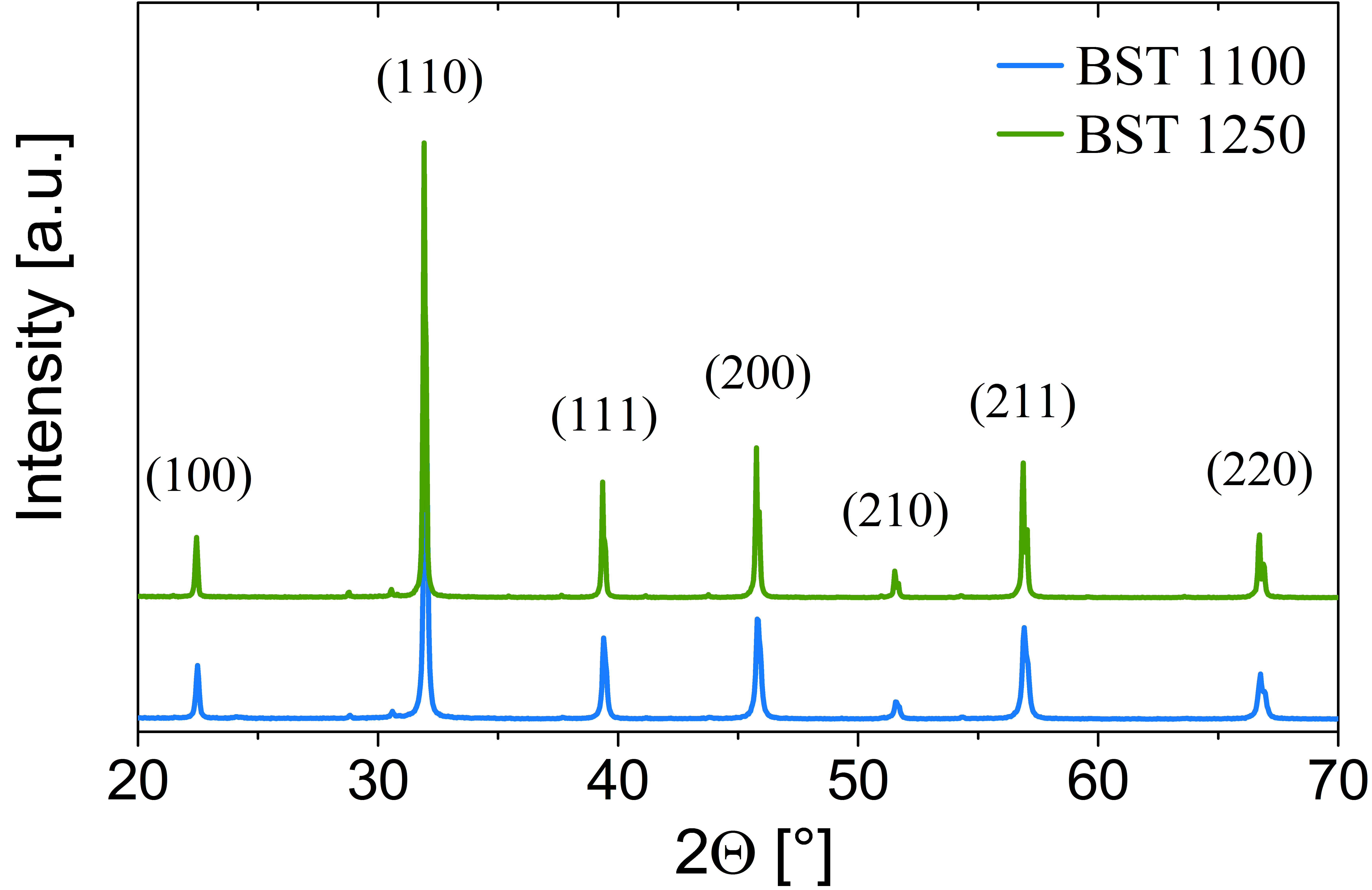

Supplement: Supplementary file 2 — Dataset S1–S7 [file 41598_2019_49639_MOESM2_ESM.zip › Fig S1.png]

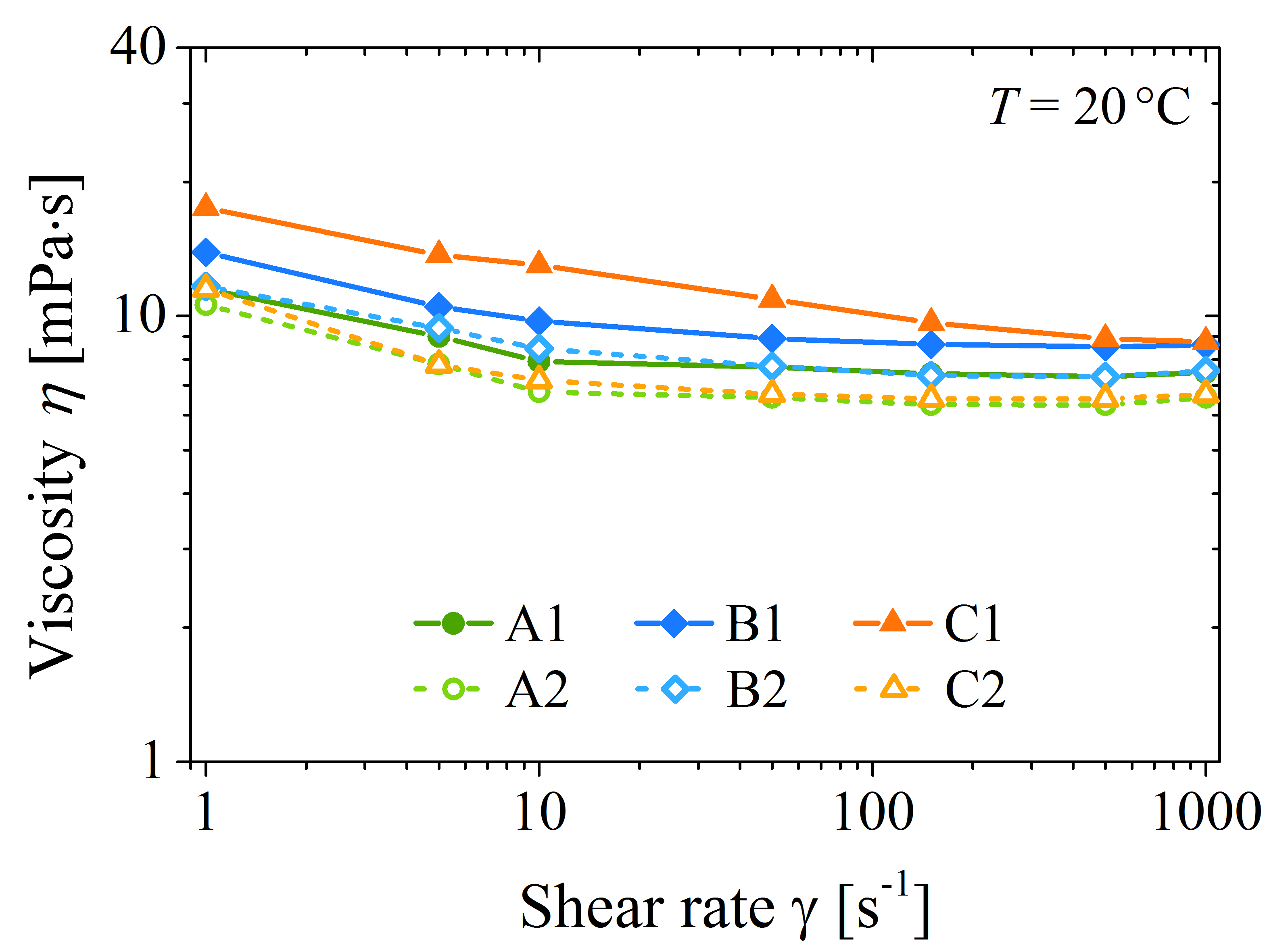

Supplement: Supplementary file 2 — Dataset S1–S7 [file 41598_2019_49639_MOESM2_ESM.zip › Fig S2.png]

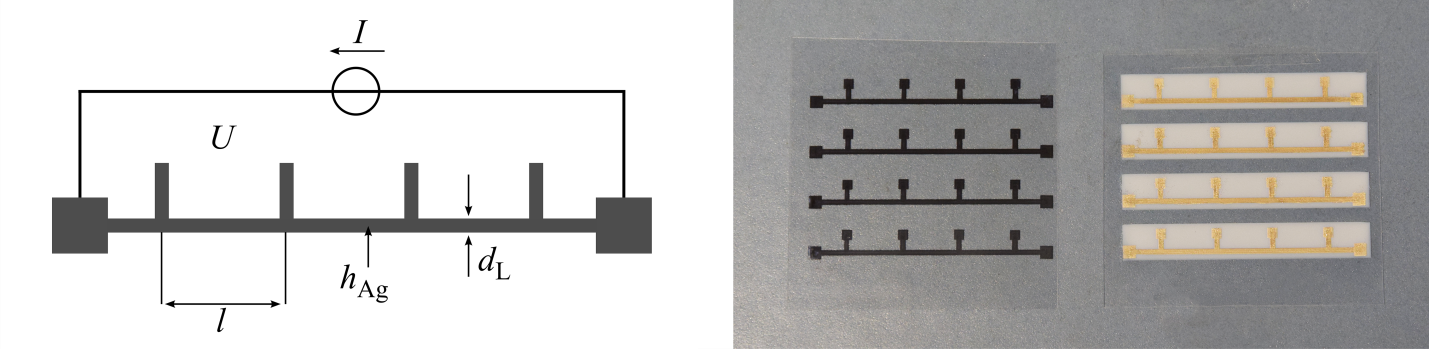

Supplement: Supplementary file 2 — Dataset S1–S7 [file 41598_2019_49639_MOESM2_ESM.zip › Fig S3.png]

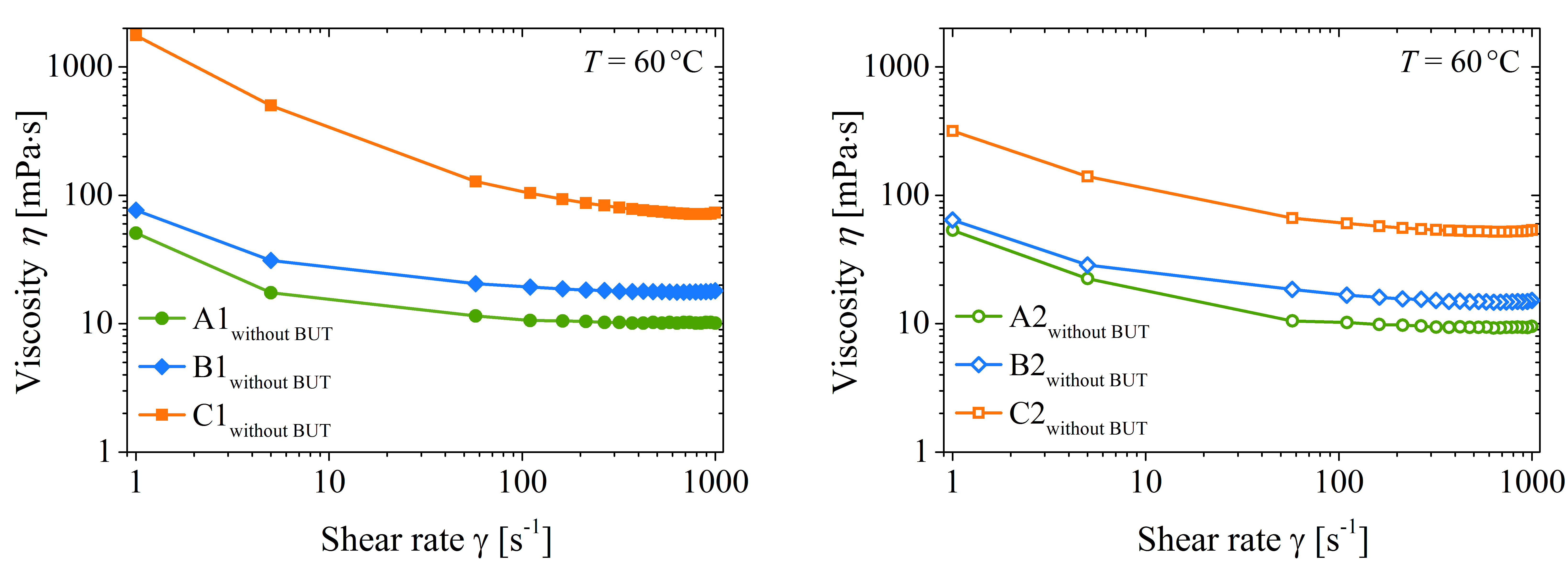

Supplement: Supplementary file 2 — Dataset S1–S7 [file 41598_2019_49639_MOESM2_ESM.zip › Fig S4.png]

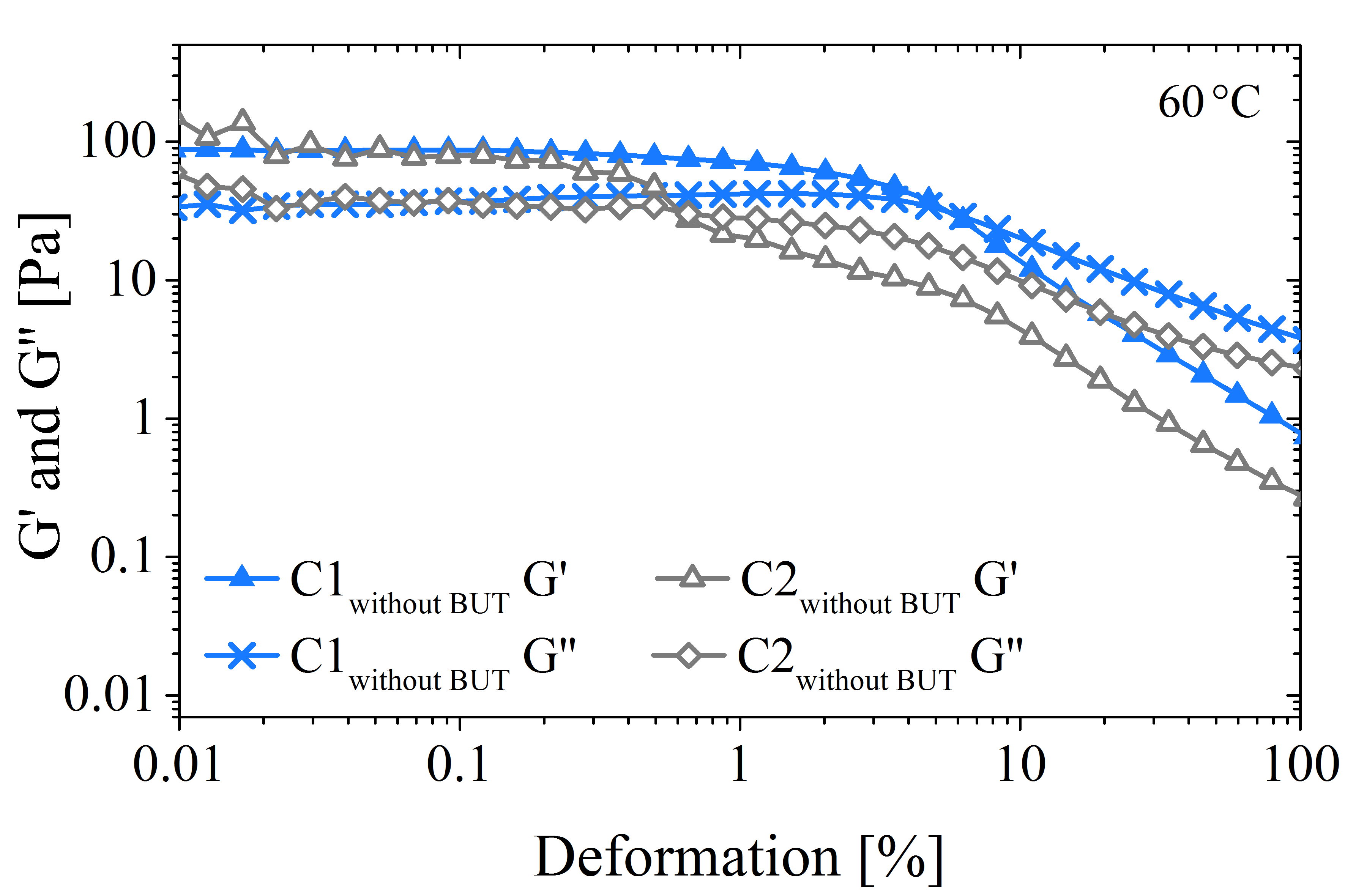

Supplement: Supplementary file 2 — Dataset S1–S7 [file 41598_2019_49639_MOESM2_ESM.zip › Fig S5.png]

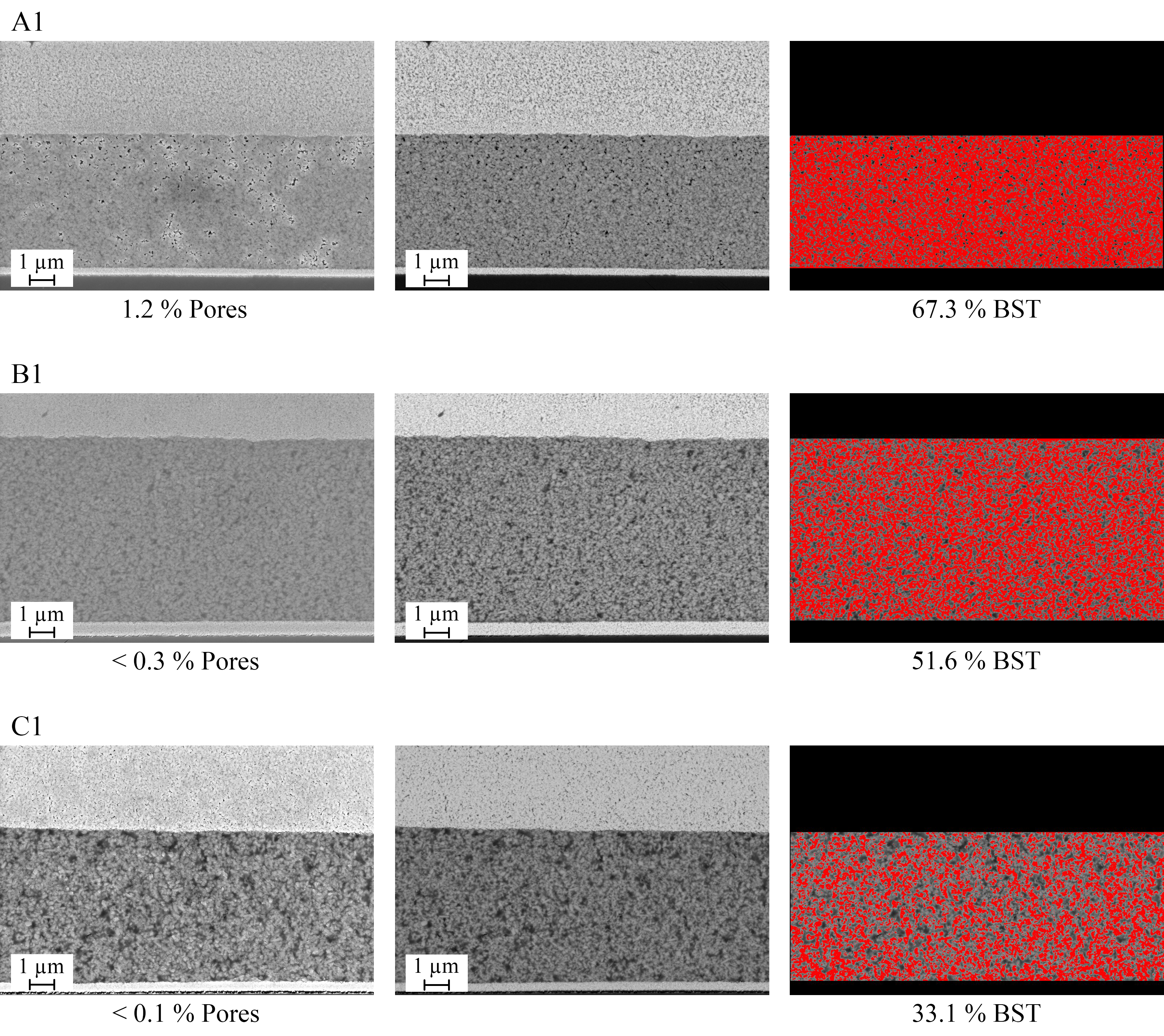

Supplement: Supplementary file 2 — Dataset S1–S7 [file 41598_2019_49639_MOESM2_ESM.zip › Fig S6.png]

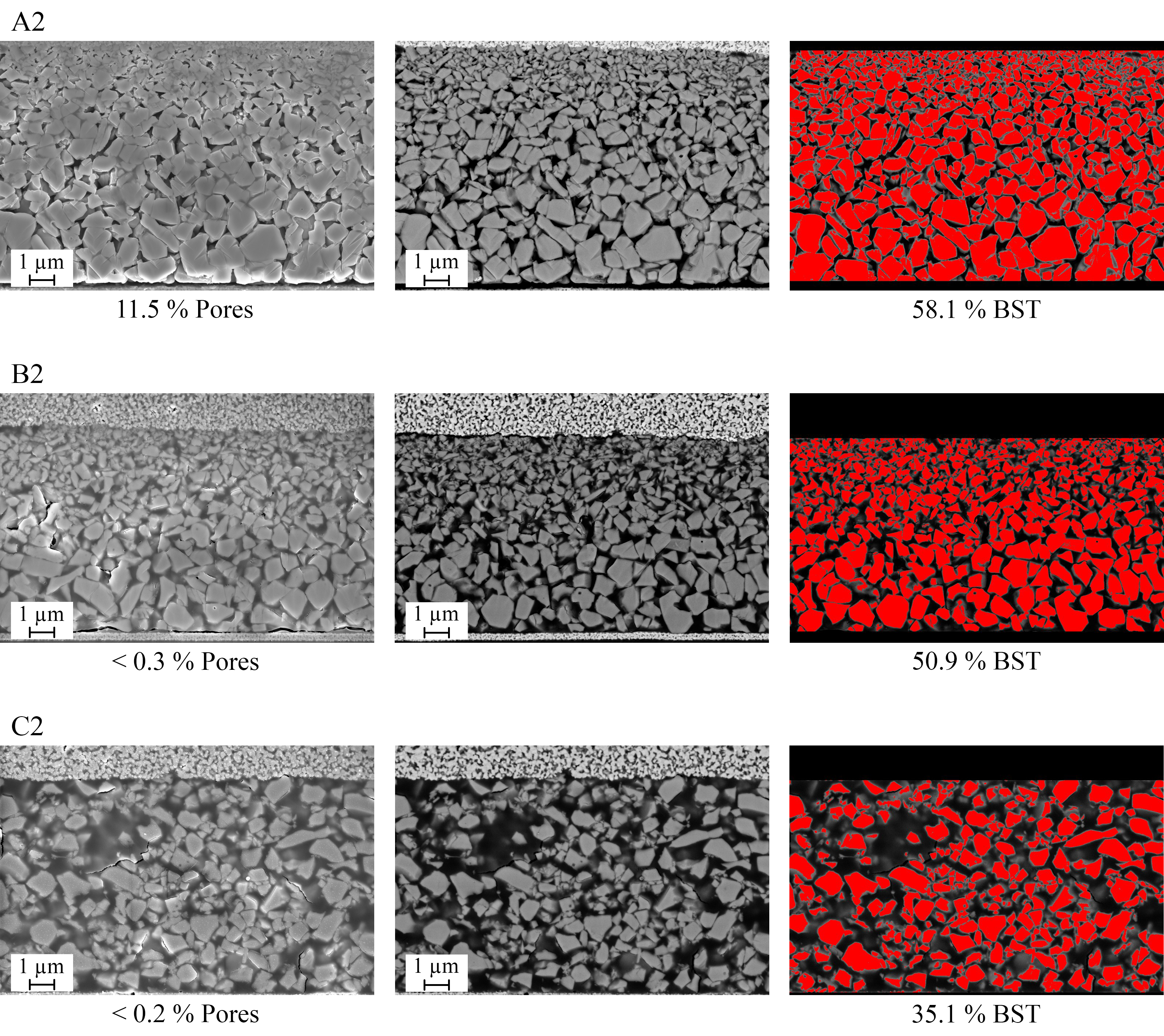

Supplement: Supplementary file 2 — Dataset S1–S7 [file 41598_2019_49639_MOESM2_ESM.zip › Fig S7.png]
